# Microglia-Mediated Inflammation and Neural Stem Cell Differentiation in Alzheimer’s Disease: Possible Therapeutic Role of K_V_1.3 Channel Blockade

**DOI:** 10.3389/fncel.2022.868842

**Published:** 2022-04-21

**Authors:** Miren Revuelta, Janire Urrutia, Alvaro Villarroel, Oscar Casis

**Affiliations:** ^1^Department of Physiology, Faculty of Medicine and Nursery, University of the Basque Country (UPV/EHU), Leioa, Spain; ^2^Instituto Biofisika, Consejo Superior de Investigaciones Científicas (CSIC)-University of the Basque Country/Euskal Herriko Unibertsitatea (UPV/EHU), Leioa, Spain; ^3^Department of Physiology, Faculty of Pharmacy, University of the Basque Country (UPV/EHU), Vitoria-Gasteiz, Spain

**Keywords:** Alzheimer’s disease, microglia, K_V_1.3, inflammation, neurodegenaration, neural stem cell (NSC), therapeutic targets

## Abstract

Increase of deposits of amyloid β peptides in the extracellular matrix is landmark during Alzheimer’s Disease (AD) due to the imbalance in the production vs. clearance. This accumulation of amyloid β deposits triggers microglial activation. Microglia plays a dual role in AD, a protective role by clearing the deposits of amyloid β peptides increasing the phagocytic response (*CD163, IGF-1* or *BDNF*) and a cytotoxic role, releasing free radicals (ROS or NO) and proinflammatory cytokines (*TNF-*α, *IL-1*β) in response to reactive gliosis activated by the amyloid β aggregates. Microglia activation correlated with an increase K_V_1.3 channels expression, protein levels and current density. Several studies highlight the importance of K_V_1.3 in the activation of inflammatory response and inhibition of neural progenitor cell proliferation and neuronal differentiation. However, little is known about the pathways of this activation in neural stem cells differentiation and proliferation and the role in amyloid β accumulation. In recent studies using *in vitro* cells derived from mice models, it has been demonstrated that K_V_1.3 blockers inhibit microglia-mediated neurotoxicity in culture reducing the expression and production of the pro-inflammatory cytokines *IL-1*β and *TNF-*α through the NF-kB and p38MAPK pathway. Overall, we conclude that K_V_1.3 blockers change the course of AD development, reducing microglial cytotoxic activation and increasing neural stem cell differentiation. However, further investigations are needed to establish the specific pathway and to validate the use of this blocker as therapeutic treatment in Alzheimer patients.

## Introduction

Alzheimer’s Disease (AD) is one of the main progressive neurodegenerative disorders and the most common cause of dementia affecting principally the elderly (Bateman et al., 2012). The histopathology is characterized by brain atrophy, deposits of amyloid β (Aβ) peptides in the extracellular matrix, neurofibrillary tangles (mainly tau protein), loss of neurons and synapses and dystrophic neurites (Hansen et al., 2018).

The increased number of Aβ amyloid plaques in the extracellular matrix, due to the imbalance in the production vs. clearance, is believed to be the principal pathogenic mechanism (Selkoe and Hardy, 2016). Familial AD is characterized by excessive production of Aβ, caused by a mutation in amyloid precursor protein (APP) or in the APP processing enzyme. However, Familial AD is extremely rare, while the majority of the AD cases are “sporadic” and occur late in life. Late AD is thought to be a result of genetic and environmental factors, and mainly aging that reduce the brain’s ability to clear Aβ (Mawuenyega et al., 2010; Wildsmith et al., 2013).

Microglial activation was initially thought to be incidental and triggered by the accumulation of amyloid deposits. Interestingly, it has been established recently that many genes found in or near AD risk loci are genes mainly expressed in microglia (Hemonnot et al., 2019). Among these genes, Apoliprotein E (*APOE*), *SP1l*, *TREM2*, or *CD33* code for proteins that are expressed principally or exclusively in microglia (Verheijen and Sleegers, 2018).

Microglia, the major inflammatory cells of the brain, play a dual role in AD. On the one hand, they play a protective role by clearing the deposits of Aβ peptides increasing the phagocytic activity (Miners et al., 2011) and on the other hand, they play a cytotoxic role by releasing cytotoxic substances and pro-inflammatory cytokines in response to reactive gliosis activated by the Aβ aggregates (Glass et al., 2010).

## Microglial Activation in Alzheimer’s Disease

**FIGURE 1:**
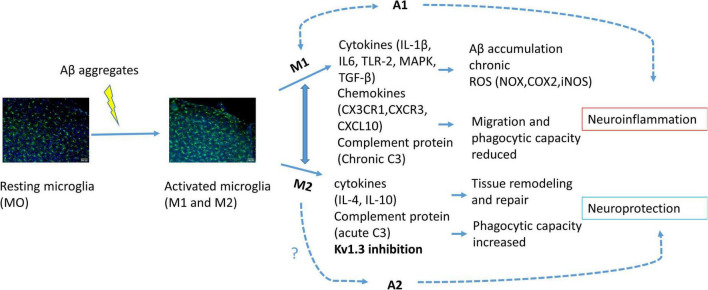
Schematic illustration of microglial activation due to Aβ accumulation. Activated microglia polarized into M1 or M2 phenotype. Activation of M1 phenotype induces pro-inflammatory cytokine, chemokine and complement protein release provoking citotoxicity and consequently astrocyte A1 activation, neuroinflammation and neuronal cell death. M2 phenotype activation induces anti-inflammatory response and consequently A2 activation and neuroprotection. K_V_1.3 inhibition has been related to M2 phenotype polarization.

Neuroinflammation is driven mostly by glial cells such as microglia and astrocytes (Forloni and Balducci, 2018). Microglia, which represent around 10–15% of human brain cells, are immune cells that first respond to nervous system changes (Kwon and Koh, 2020; Liu et al., 2021). Microglia are categorized mainly in two opposite phenotypes depending on their specific markers that define cell type and state; the pro-inflammatory M1 (classical activation) and M2 (alternative activation) phenotypes (Mills et al., 2000; Bi et al., 2021). Depending on the activated phenotype, microglia can produce either cytotoxic or neuroprotective effects. The classical activation is related to pro-inflammatory cytokine production, such as tumor necrosis factor-α (*TNF-*α), interleukin-1β (*IL-1*β) and reactive oxygen species (ROS) or nitric oxide (NO) production. Meanwhile, the alternative activation promotes anti-inflammatory response with increased *IL-4, IL-10, CD36* and phagocytic response expressing CD163, insulin-like growth factor 1 (IGF-1) and brain derived neurotrophic factor (BDNF) (Le et al., 2016; Chen et al., 2021; Figure 1). The microglial polarization has not been supported by single-cell RNA-seq, while this transcriptomic analysis have been used to distinguish between diseased associated microglia (DAM) or activated response microglia (ARM) (Keren-Shaul et al., 2017; Sala Frigerio et al., 2019). Transcriptome data show that during neurodegenerative diseases both phenotypes, the neurotoxic and the neuroprotective, are expressed (Sarlus and Heneka, 2017; Figure 1).

During AD, microglial activation is associated with Aβ deposits in human and mice brains (Kamphuis et al., 2012; Olmos-Alonso et al., 2016). Indeed, activated microglia in AD mouse models express increased inflammatory markers *CD36, CD14, CD11c*, major histocompatibility complex-II (*MHC-II*), and inducible nitric oxide synthase (*iNOS*), as well as M1 phenotype markers.

The inflammatory response is typically composed of three main stages. First, toll-like receptor (TLR)-mediated NF-κB formation. This leads to an increase of mainly members of the nod-like receptor (NLR) that assemble the inflammasome that third, activates caspase-1, which cleaves the precursor protein and release *IL-1*β (Yin et al., 2016).

While anti-inflammatory cytokines may have a deleterious role in AD (Guillot-Sestier et al., 2015), TLR activation and *IL-1*β secretion may also have protective effects (Shaftel et al., 2007; Richard et al., 2008).

### Cytokines

Post-mortem immunohistochemical studies of brain tissues show that among all cytokines that are highly expressed during AD, *IL-1*β, *IL-6*, and *TNF-*α are the most abundant (Bernhardi et al., 2015). Increased levels of these cytokines may play different roles in the context of Aβ deposition. Remarkably, *IL-1*β, a key cytokine of innate immune response, enhance Aβ and tau pathology (Lee et al., 2013), while increased levels of *TNF-*α may facilitate the Aβ clearance (Montgomery et al., 2011; Sarlus and Heneka, 2017).

Particularly, *IL-1*β activates astrocytes that may contribute to plaque formation due to the release of astrocyte-derived proteins, such as *IL-6, APOE* and some complement proteins. Furthermore, *IL-1*β induces neurite growth promoting the cytokine S100β. *S100*β induces the increase of Aβ precursor protein, so it has been linked with the initial deposition of Aβ (Griffin and Mrak, 2002). The abnormal accumulation of Aβ plaques also triggers the excessive release of other anti-inflammatory cytokines, such as *IL-4, IL-10, IL-13* that accelerates tissue remodeling, repair and angiogenesis and inhibits the production of other pro-inflammatory cytokines (Stamouli and Politis, 2016; Kaur et al., 2019).

During neuroinflammation in AD there is also an activation of TLR-2. This activation triggers the nuclear translocation of NF-κB and provokes Aβ-induced inflammation and chronicity of AD (Zhao et al., 2013).

Some protein kinases such as mitogen-activated protein kinase (MAPK), cell division cycle 2 kinase (CDC2) and Janus kinase-signal transducer and activator of transcription (JAK-STAT) pathways have been also identified in AD progression (Rather et al., 2021). Activated MAPK and NF-κB increase the production of pro-inflammatory cytokines promoting APP processing, blood-brain barrier (BBB) disintegration and aggravates tau protein phosphorylation. Moreover, the formation of neurofibrillary tangles due to p38-MAPK activation leads to neuronal degeneration and finally neuronal death (Jeong et al., 2014; Rather et al., 2021).

### Chemokines

Chemokines, a large family of small (8–14 kDa) basic proteins, are also important inflammatory mediators overexpressed during inflammatory events in the CNS. During AD, several chemokines have been associated with microglia activation due to Aβ depositions. For example, CCR2 (C-C motif chemokine receptor type 2) is a chemokine expressed on microglia that accumulates mononuclear phagocytes in inflammatory sites. Studies show that lack of CCR2 decreases microglial accumulation and results in an increased Aβ deposition, indicating that CCR2 may play a protective role in AD promoting Aβ clearance (El Khoury et al., 2007).

Moreover the lack of CCR2 stimulates the expression of *TGF-*β and *CX3CR1* (CX3C chemokine receptor 1) in microglia (Guedes et al., 2018). Interestingly, several murine AD mice models revealed that genetic elimination of *CX3CR1*, a chemokine receptor predominantly found in microglia, resulted in a decrease of amyloid plaques due to the increase of phagocytic capacity in the activated microglia (Guedes et al., 2018).

### Complement Proteins

The complement system, composed of about 30 proteins, plays an important role in host defense and in the inflammatory regulation (Crehan et al., 2012). The accumulation of Aβ plaques and increased neurofibrillary tangles activate the classical complement pathway in microglia within the collagen-like domain of C1q (Shen et al., 2001). Nevertheless, complement’s role needs to be further studied (Rasmussen et al., 2018), as C3, a central component in the activation of the complement system, provokes different responses to microglial phagocytosis.

The complement-dependent mechanism can also mediate synapse loss by swallowing this synapse. During AD, this synapse loss involves a pathway in which the complement clears pathogens and apoptotic cells after binding of complement protein C1q. Thus, blocking microglial activation or the activation of complement mechanism may have beneficial effects in AD reducing synapse and neuronal loss (Hansen et al., 2018).

### Free Radicals

Some authors described that the abnormal accumulation of Aβ and the deposition of neurofibrillary tangles extend oxidative damage, impair Ca^2+^ homeostasis and produce mitochondrial dysfunction during AD (Bello-Medina et al., 2021). Nevertheless, other studies claim that during AD the increased ROS production and altered Ca^2+^ homeostasis precede Aβ accumulation and is due to mitochondrial dysfunction (Yoo et al., 2020). Anyway, increased microglial ROS production contributes to oxidative stress resulting in neuronal dysfunction and neurotoxicity. Moreover, microglia respond to damage-associated molecular patterns (DAMPs) released from damaged cells, activating NADPH oxidase (NOX). In fact, the activation of the phagocyte NOX2 in microglia seems to play an important role in neuroinflammation and in neuronal death (Qin et al., 2013; Jiang et al., 2015).

Microglia produce pattern recognition receptors (PRR) also in response to DAMPs stimuli, such as Complement receptor 3 (CR3) or TLR. These PRR mediate activation of pro-inflammatory signaling traducers NLRP3 inflammasome, NF-κB and MAPKs (Simpson and Oliver, 2020).

## Microglial Ion Channels and Alzheimer’s Disease

In healthy brains, microglia regulate the correct development and function of synapses and synaptic plasticity. Microglia-synapse disruption may contribute to synapse loss, dysfunction and, consequently, disease (Hong et al., 2016). There are many studies concerning the effect of changes in cytokines, chemokines or ROS production in microglial activation. However, little is known about the effects of changes in the intracellular ionic homeostasis and microglial activation (Izquierdo et al., 2019). Ion channels are involved in many microglial functions, such as cytokine production, migration, production or proliferation, among others.

Microglia membrane express different ion channels, such as Ca^2+^-, K^+^-, Na^+^-, H^+^- and Cl^–^-channels, in order to face all physiological functions. For instance, Ca^2+^-channels are important for intracellular Ca^2+^ homeostasis in microglia. Store-operated Ca^2+^-channels, voltage-gated Ca^2+^-channels and transient receptor potential channels control Ca^2+^ signaling for microglial activation (Luo et al., 2021). Inward rectifier channels and voltage-gated K^+^-channels as well as Cl^–^-channels (volume regulated Cl^–^-channels and chloride intracellular channels), are not only necessary for cell hyperpolarization, but for cell activation and proliferation. This is because they supply the driving force that allow an increase intracellular Ca^2+^ concentration *via* Ca^2+^-channels (Nguyen et al., 2017).

Voltage-gated and acid-sensing Na^+^-channels are also relevant in these non-excitable cells to regulate cell migration, phagocytosis, and secretion of cytokines (Pappalardo et al., 2016). Finally, voltage-gated H^+^-channels are also important to regulate cells’ pH.

Glial cells express the voltage-gated K^+^-channels K_V_1.3 and K_V_1.5. These channels activity changes in microglial activation by modifying their relative expression. In fact, one characteristic of activated inflammatory cells is an increased expression and function of the K_V_1.3 channels (Pérez-Verdaguer et al., 2016). In quiescent cells K_V_1.5 regulates the proliferation rate (Pannasch et al., 2006; Gubiè et al., 2021) while K_V_1.3 is dominant in activated microglial cells (Gubiè et al., 2021). Besides, K_V_1.5 seems to be essential for NO production (Pannasch et al., 2006), but causes cell cycle arrest. On the other hand, K_V_1.3 participates in microglial proliferation and migration, as well as in the cytokine release (Charolidi et al., 2015; Stebbing et al., 2015). LPS-mediated microglial activation induces cytokine release, but decreases proliferation (Pannasch et al., 2006). In response to Aβ accumulation K_V_1.3, K_V_1.5 and calcium-activated K^+^-channels (KCa3.1, KCa2.3, or BK channels) increase voltage-dependent Ca^2+^ entry provoking a disruption in Ca^2+^ homeostasis and consequently neurodegeneration (Dolga et al., 2012; Kumar et al., 2016; Huang et al., 2021). Among all these channels, the most studied is K_V_1.3.

### K_V_1.3 in Microglia During Alzheimer’s Disease

Within all type of microglial channels, K_V_1.3 has a fundamental role in the activation of these cells, since it contributes to maintaining the negative membrane potential. This channel is a Shaker-type voltage-gated K^+^-channel with six transmembrane domains (Wulff and Zhorov, 2008) and it is widely distributed throughout the whole body, being highly expressed in both nervous and immune systems. First described in T cells (DeCoursey et al., 1984), it has been related with autoimmune diseases mostly as it plays an important role in immune cell activation by modulating Ca^2+^ signaling (Wulff et al., 2007; Feske et al., 2015).

**FIGURE 2:**
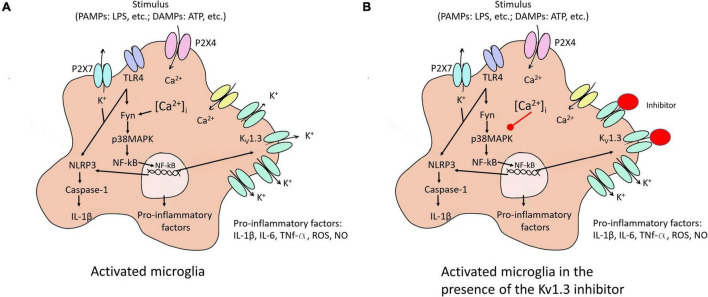
Microglia activation to M1 and the K_V_1.3 blockade effect on this activation. **(A)** Different stimuli activate microglia to pro-inflammatory state (M1). The activation of the NF-κB pathway induced an increase of K_V_1.3 protein in the membrane, among others effects. **(B)** K_V_1.3 inhibitor provokes a smaller Ca^2+^ entry reducing secretion of pro-inflammatory factors due to the decrease in the activation of NF-κB pathway. P2 × 7 and P2 × 4, purinergic receptors; TLR4, toll like receptor 4; NLRP3, NOD-, LRR- and pyrin domain-containing protein 3; PAMPs, exposure to pathogen-associated molecular patterns; DAMPs, endogenous damage-associated molecular patterns; ROS, reactive oxygen species; Fyn, non-receptor tyrosine-protein kinase; IL, interleukin; TNF, tumor necrosis factor; iNOS, nitric oxide synthase, NO, nitric oxide.

Microglia activation provokes an overexpression of K_V_1.3 mRNA and protein levels, which lead to increased current densities (Nguyen et al., 2017). K_V_1.3 is required for microglial pro-inflammatory activation and neurotoxicity (Figure 2A) and is highly expressed by microglia in human AD brains and AD mice models (Rangaraju et al., 2015).

Overexpression of K_V_1.3 channels in a microglial cell line increases the expression and secretion of different interleukins (Sarkar et al., 2020). Mice exposed to experimental stroke (Chen et al., 2016) as well as treatment with lipopolysaccharide (LPS) or a combination of LPS and IFN-γ in the microglia of mouse models (Nguyen et al., 2017; Di Lucente et al., 2018) exhibit increased K_V_1.3 current in affected areas. LPS-mediated microglial activation induces cytokine release, but decreases proliferation (Pannasch et al., 2006). In addition, stimulus such LPS or ATP activates the NF-κB pathway *via* different membrane receptors [purinergic receptor (P2 × 4 and P2 × 7) and TLR4] leading to an overexpression of K_V_1.3 and pro-inflammatory factors provoking neuroinflammation (Figure 2A). Concerning this, Di Lucente et al. (2018) demonstrated that blockade K_V_1.3 blockade after LPS treatment induces M2 microglia polarization reducing pro-inflammatory markers.

ATP and the increased K^+^ efflux augment Ca^2+^ entry, which raises the inflammatory state of the cell through the activation of NF-κB pathway *via* p38MAPK phosphorylation. At the same time, NF-κB interacts with their binding sites in the K_V_1.3 promotor. All these changes cause NLRP3 inflammasome activation conducting *IL-1*β secretion (Figure 2A). Besides, inhibition of NF-κB or up-regulation of K_V_1.3 provoked by αSynAgg stimulation indicates that both p38MAPK and NF-κB pathways intervene in the transcriptional regulation of the channel (Sarkar et al., 2020).

## Effect of Microglia on Neural Stem Cell Differentiation in Alzheimer’s Disease

There is a controversy about human neurogenesis. Some authors conclude that hippocampal neurogenesis is extremely rare in the adult brain, as they did not detect new neurons in the dental gyrus (Sorrells et al., 2018). However, other authors observed immature neurons, neuroblast and neural progenitor cells in aged human hippocampus (Boldrini et al., 2018; Tobin et al., 2019).

In the adult mammalian brain, neural stem cells (NSCs) are localized in two major neurological niches, the subgranular zone of the hippocampus (SGZ) and the subventricular zone of the lateral ventricle (SVZ). These cells retain the ability to proliferate and differentiate into neurons and glial cells (Moreno-Cugnon et al., 2019).

Some studies report that microglia in the hippocampus are more active than in other brain regions, playing an important role in refining neuronal circuits (Rao et al., 2022). Furthermore, microglia release several cytokines that promote microglial migration, neuroblast generation and neurogenesis and is considered a crucial component for determine NSC fate (Shigemoto-Mogami et al., 2014; Geribaldi-Doldán et al., 2021).

The hippocampus is one of the most affected brain regions in AD with altered dentate granule cells. There have been several studies using NSC/induced pluripotent stem cells (iPSCs) derived from Alzheimer patients with the objective of promoting neurogenesis and ameliorating the progression of the disease (Wu et al., 2021). In one of these studies, for example, they demonstrate that the release of pro-inflammatory cytokines, such as *TNF-*α, *IL-1*β, and *IGF-1* by microglia enhance the dopaminergic differentiation of neural stem cells and promote neurogenesis (Boyd et al., 2021; Schmidt et al., 2021).

As mentioned, impaired *CX3CR1* has been described in AD (Guedes et al., 2018). In the hippocampus, this impairment has been linked to adult hippocampal neurogenesis disruption, with spatial and fear-memory and motor learning loss due mainly to the increase of *IL-1*β by microglial activation (Parkitny and Maletic-Savatic, 2021). Remarkably, the importance of *IL-1*β in adult hippocampal neurogenesis was recognized over a decade ago when it was associated with anti-proliferative and anti-neurogenic effects (Crampton et al., 2012). Moreover, PRR seem to be implicated in the modulation of adult neurogenesis as they are expressed also in neural progenitor cells (NPCs), providing communication pathways from apoptotic or injured cells (Parkitny and Maletic-Savatic, 2021).

### Role of K_V_1.3 in Neural Stem Cell Differentiation

Recent studies support the hypothesis that cell proliferation and division depend on K^+^-channels activity (Gallo et al., 1996; Jäger and Grissmer, 2004). K_V_1.3 channels control action potential firing of hippocampal and OB neurons, representing around 60–80% of all K^+^-channels in these areas (Martínez-Mármol et al., 2016). This channel has also been found in NPC. Moreover, Liebau et al., 2006 demonstrated that blockade of K_V_1.3 by Psora-4 [5-(4-Phenylbutoxy) psoralen] increases the number of NPC *in vitro*. In 2010, Wang et al. (2010) also exposed the importance of K_V_1.3 in the activation of inflammatory response and inhibition of NPC proliferation and neuronal differentiation. However, little is known about the pathways of this activation in NSC differentiation and proliferation.

## K_V_1.3 as a Potential Therapeutic Target in Alzheimer’s Disease

To date, there is no treatment to cure or prevent AD. Current treatments are only useful in slowing down the progression of the disease and in managing some behavioral and cognitive symptoms of AD patients. Because of the complex pathophysiology, sometimes the treatment needs to be a combination of therapies. Currently, only six treatments are approved in the US. These include three cholinesterase inhibitors (donepezil, galantamine, and rivastigmine), one N-methyl-D-aspartate receptor antagonist (memantine) (Cummings et al., 2019), a fixed-dose combination with donepezil and memantine and finally, the recently approved aducanumab, a human monoclonal antibody that targets, and reduces Aβ accumulations in the brain.

As mentioned, during AD there is a release of cytotoxic substances and pro-inflammatory cytokines by the M1 activation provoking neuronal damage and aggravating AD pathology (Heneka et al., 2015). In this M1 activated state, K_V_1.3 channels are upregulated. Little is known about the mechanism in which these channels are activated and the consequences in AD. Recent studies have analyzed the effect of K_V_1.3 blockers on microglial profiles in AD models and confirm that pro-inflammatory and neurotoxic microglia functions are reduced with different K_V_1.3 inhibitors (Figure 2B). K_V_1.3 blockade decreases cerebral amyloid load, enhances hippocampal neuronal plasticity, and improves behavioral deficits by a reduction of microglia activation and inflammatory cytokines levels in transgenic AD mouse models (Chen et al., 2018; Maezawa et al., 2018; Ma et al., 2020). Accordingly, K_V_1.3 blockers inhibit microglia-mediated neurotoxicity in culture (Fordyce et al., 2005) and protect mice from microglia-mediated radiation-induced brain injury *in vivo* (Peng et al., 2014).

Classification of K_V_1.3 channel blockers depends on their selectivity and blockade potency. The most effective inhibitors are the natural peptides such as the sea anemone *Stichodactyla helianthus* toxin ShK and scorpion toxins HsTx, OSK1 and Vm24. These molecules present high affinity for different channels. An analog of Shk, the ShK-223, diminished the activity of the pro-inflammatory microglia and elevated Aβ clearance in AD models (Rangaraju et al., 2018; Ramesha et al., 2021). HsTX1 [R14A] mutant, is a potent, selective and highly stable peptide inhibitor. It has been shown that this inhibitor reduces the release of *TNF-*α and *IL-6* by LPS-mediated BV-2 microglia activation improving neuroinflammation (Nicolazzo et al., 2022).

Beside these peptides, there are different synthetic organic small-molecules inhibitors such as, PAP−1 [5-(4-phenoxybutoxy) psoralen], Psora−4, dihydroquinoline, benzamides, clofazimine, furoquinoline, acridinone, furochromene−7−thione, diphenoxylate, and several analogs are used. All these inhibitors differ in the potency to block K_V_1.3 channel and the selectivity for the target (Gubiè et al., 2021).

In rodents, PAP-1 and ShK-223 reduce the expression and production of several cytokines (*IL-1*β, *IL-4, IL-5, IL-10, IL-12, IFN*γ and *TNF-*α) (Nguyen et al., 2017; Zayas-Arrabal et al., 2021) and decreased Aβ plaque burden in the 5xFAD mice brain. Furthermore, these molecules increased Aβ phagocytosis by microglia and not blood derived monocytes due to the K_V_1.3 channel blockade (Maezawa et al., 2018; Ramesha et al., 2021). The pharmacological blockade of the channel in AD mice model promotes synaptogenesis and polarizes microglial phenotype toward M2 (Ramesha et al., 2021).

However, the use of pharmacological K_V_1.3 blockers may have several adverse effects. ShK and HsTX1 blockers, for instance, have difficulties to penetrating the intestinal mucosa so they can’t be taken orally and they neither can cross the BBB. Therefore, there is a need for cell penetrating peptides to assist the passage of the drug across the cell membrane (Wang et al., 2020). Other problem relates to the selective of K_V_1.3 blockade. It is important to mention that K_V_1.3 is also expressed in mitochondria. This channel controls cell-proliferation and has an important role in cellular respiration (Styles et al., 2021). Thus, the use of some small-molecule K_V_1.3 blockers as therapy could also block mitochondrial channels, inducing apoptosis in cancer cells (Teisseyre et al., 2019). According to recent studies, the most potent and selective small-molecule K_V_1.3 inhibitor available is PAP-1 (Peruzzo et al., 2020).

However, PAP-1, PSORA-4 and some derivatives produce apoptosis in cancer cell lines. These blockers inhibit K_V_1.3 channels both at the plasma and mitochondrial membranes, causing an increased ROS production and, finally, apoptosis. The activation of the apoptotic pathway by these inhibitors is mainly due to cancer cells’ massive ROS release (Checchetto et al., 2019). Therefore, the therapeutic use of these inhibitors in AD may induce microglial apoptosis by further increasing the cell’s basal level of ROS. Additional studies need to be performed in order to determine the cytotoxicity of this K_V_1.3 channel blocker in AD.

In summary, K_V_1.3 plays important roles in regulating membrane potential, preventing depolarization and controlling Ca^2+^ signaling events reducing microglia activation. However, further investigation is needed to achieve a deeper understanding of the role of K_V_1.3 in the microglial immune response and to identify specific pathways for enhancement of Aβ plaque formation or NPC differentiation. Moreover, the potential pharmacological use of drugs targeting K_V_1.3 channels requires further characterization.

## Author Contributions

MR and JU wrote the article. AV and OC reviewed. All authors contributed to the article and approved the submitted version.

## Conflict of Interest

The authors declare that the research was conducted in the absence of any commercial or financial relationships that could be construed as a potential conflict of interest.

## Publisher’s Note

All claims expressed in this article are solely those of the authors and do not necessarily represent those of their affiliated organizations, or those of the publisher, the editors and the reviewers. Any product that may be evaluated in this article, or claim that may be made by its manufacturer, is not guaranteed or endorsed by the publisher.
